# *Escherichia coli* and *Candida albicans* Induced Macrophage Extracellular Trap-Like Structures with Limited Microbicidal Activity

**DOI:** 10.1371/journal.pone.0090042

**Published:** 2014-02-25

**Authors:** Pan Liu, Xiuping Wu, Chengshui Liao, Xiaolei Liu, Jing Du, Haining Shi, Xuelin Wang, Xue Bai, Peng Peng, Lu Yu, Feng Wang, Ying Zhao, Mingyuan Liu

**Affiliations:** 1 Key Laboratory for Zoonosis Research, Ministry of Education, Institute of Zoonosis, Jilin University, Changchun, China; 2 Mucosal Immunology Laboratory, Pediatric Gastroenterology Unit, Massachusetts General Hospital East, Charlestown, Massachusetts, United States of America; 3 Jiangsu Co-innovation Center for Prevention and Control of Important Animal Infectious Diseases and Zoonoses, Yangzhou, China; The Hospital for Sick Children and The University of Toronto, Canada

## Abstract

The formation of extracellular traps (ETs) has recently been recognized as a novel defense mechanism in several types of innate immune cells. It has been suggested that these structures are toxic to microbes and contribute significantly to killing several pathogens. However, the role of ETs formed by macrophages (METs) in defense against microbes remains little known. In this study, we demonstrated that a subset of murine J774A.1 macrophage cell line (8% to 17%) and peritoneal macrophages (8.5% to 15%) form METs-like structures (METs-LS) in response to *Escherichia coli* and *Candida albicans* challenge. We found only a portion of murine METs-LS, which are released by dying macrophages, showed detectable killing effects on trapped *E. coli* but not *C. albicans*. Fluorescence and scanning electron microscopy analyses revealed that, *in vitro,* both microorganisms were entrapped in J774A.1 METs-LS composed of DNA and microbicidal proteins such as histone, myeloperoxidase and lysozyme. DNA components of both nucleus and mitochondrion origins were detectable in these structures. Additionally, METs-LS formation occurred independently of ROS produced by NADPH oxidase, and this process did not result in cell lysis. In summary, our results emphasized that microbes induced METs-LS in murine macrophage cells and that the microbicidal activity of these METs-LS differs greatly. We propose the function of METs-LS is to contain invading microbes at the infection site, thereby preventing the systemic diffusion of them, rather than significantly killing them.

## Introduction

Macrophages are dynamic cells distributed in various tissues that play important roles in immune processes. These cells are well known for their ability to eliminate various microbial pathogens through phagocytosis. A combination of oxidative and non-oxidative microbicidal mechanisms is employed to the killing of phagocytosed microbes, including the production of antimicrobial peptides/degradative enzymes, and the generation of antimicrobial reactive oxygen species (ROS)/nitric oxide synthase (iNOS) [1], [2]. However, these mechanisms are not effective in defense against microbes that have either evolved various strategies that interfere with phagocytosis or that are too large to be engulfed (e.g., fungal hypha) [3], [4].

Recently, a phagocytosis-independent antimicrobial mechanism, described as extracellular traps (ETs), was identified in many innate effector cells including neutrophils, mast cells, eosinophils and macrophages [5], [6], [7], [8]. ETs are fiber-like extracellular structures that can be induced by many different microbes. These structures have been implicated as a physical barrier and an additional defense strategy in response to infections. Studies on neutrophil extracellular traps (NETs) have revealed that this mechanism exists in many species, including human, mice, cows, horses and fish [5], [9], [10], [11], [12]. NETs are composed of DNA scaffolds “decorated” with antimicrobial proteins, such as histone and granule proteins (e.g., myeloperoxidase, elastase, cathelicidins, cathepsin G and gelatinase B) [5], [13], [14]. This organization enables neutrophils to release active effector molecules into the extracellular space and prevent local diffusion, thus preventing the potential toxicity to the host [15]. The release of these effector molecules may also enhance local concentrations of these molecules and promote their synergy [15]. NETs bind and kill several types of microbes such as the bacteria *Escherichia. coli*, *Shigella flexneri*, *Staphylococcus aureus*, the fungi *Candida albicans*, *Aspergillus fumigatus* and the protozoan parasites *Leishmania amazonensis* and *Eimeria bovis* [5], [16], [17], [18], [19], [20]. The ETs released from eosinophils and mast cells also effectively kill microbes, including *E. coli*, *S. aureus*, *Streptococcus pneumoniae* and *Sreptococcus pyogenes* [6], [7].

In addition to microbes, many chemical reagents and cytokines also induce the formation of ETs. Among these, the protein kinase C activator phorbol-12-myristate-13-acetate (PMA) is the most commonly used stimulus. Similar to microbes, PMA induces the release of ETs from neutrophils and mast cells, which contain nuclear DNA components in a process that can cause cell death [21]. This novel cell death program, designated as “NETosis”, showed characteristics of neither apoptosis or programmed necrosis [21]. In addition, viable neutrophils and eosinophils generate ETs following stimulation with the pro-inflammatory cytokines, such as GM-CSF/LPS or GM-CSF/C5a; these ETs contain mitochondrial, rather than nuclear, DNA components [6], [22]. The NADPH oxidase enzyme complex-mediated production of ROS was reported to be essential for the initiation of both types of ETs [22], [23], [24]. However, recently studies have demonstrated that NETs are also induced in a ROS-independent manner [16], [25], [26], and that the ROS requirement for these processes depends on the type of stimulus [27].

While many studies have been performed to evaluate the role NETs played in an array of activities, very little effort has been devoted on studying METs. Recently, it has been reported that murine macrophage cell line RAW264.7 and peritoneal macrophages released METs in response to PMA stimulation after pretreatment with statins overnight [8]. RAW264.7 macrophages also form METs in response to TNF-α [28]. Bovine macrophages produce METs within minutes after stimulation with *Histophilus somni* or the leukotoxin of *Mannheimia haemolytica* with a ROS-dependent manner [29], [30]. In these studies, the histones were observed to co-localize with extracellular DNA, implying that the DNA in METs may originate from the nucleus [8], [29]. Moreover, macrophages differentiated from human peripheral blood monocytes also release METs after challenge with *Mycobacterium tuberculosis*, and this effect could be enhanced by pre-treating macrophages with IFN-γ [31]. Although METs have been reported to be triggered by several stimuli and are capable to reduce several bacteria, some features of METs such as the origination of these structures and whether they really kill microbes remain less well defined. In the present study, we demonstrate that both bacteria (*E. coli*) and fungi (*C. albicans*) induce murine METs-like structures (METs-LS) formation and that *in vitro,* these METs-LS are capable of capturing the microbes but not efficient in killing them. Further, our results on J774A.1 cells reveal that the microbe induced METs-LS show more than one type of composition, which are released from macrophages in a NADPH oxidase-independent manner.

## Materials and Methods

### Bacterial and Fungal Strains

*E. coli* strain ATCC 25922 and *C. albicans* strain ATCC 10231 were obtained from the American Type Culture Collection (ATCC, Manassas, VA, USA). *E. coli* was cultured in Luria-Bertani (LB) broth at 37°C to the mid-log phase. *C. albicans* was cultured overnight in Yeast Extract Peptone Dextrose (YPD) broth at 30°C, and an aliquot was subcultured in fresh YPD broth for an additional 4 h at 30°C. Both microbes were collected through centrifugation at 6,000 *g* for 5 min, washed twice with cold sterile PBS and diluted to the required concentration before co-incubating with macrophages. For some experiments, GFP-labeled *E. coli* 25922 was constructed by transforming pCN57 plasmid (kindly provided by Dr. Richard P. Novick, NYU Medical Center) and cultured as described above.

### Cells Cuture

Murine J774A.1 macrophage cell line was obtained from the ATCC. Cells were maintained in Dulbecco’s Modified Eagle Medium (DMEM, Gibco-Life Technologies, Grand Island, NY, USA) supplemented with 10% heat-inactivated FCS, 2 mM L-glutamine and 100 U/ml penicillin/100 µg/ml streptomycin (Invitrogen, Carlsbad, CA, USA). Cells were cultured at 37°C with 5% CO_2_ in a humidified incubator.

Thioglycollate-elicited murine peritoneal macrophages were prepared by peritoneal lavage. Briefly, 1 ml of Brewer’s complete thioglycollate broth (Difico, Sparks, MD, USA) was intraperitoneally (i.p.) injected into female BALB/c mice 4 days before cell collection. The mice were sacrificed through cervical dislocation, and their abdominal cavities were washed with 10 ml of DMEM medium. Lavage fluids were recovered and centrifuged for 10 min at 400 *g*, and cell pellet was resuspended in fresh DMEM to an appropriate concentration. The cell suspension was subsequently placed into the wells of a cell culture plate or glass bottom dishes and incubated for 2 h. Non-adherent cells were removed after washing 5 times with warm PBS. Fresh DMEM was added to the wells and cells were used for the corresponding experiments. Murine peritoneal macrophages isolation protocols were approved by the Jilin University Animal Care and Use Committee.

### Fluorescence Microscopy

Macrophages were seeded onto 12-mm glass cover slides in 24 well plate (2×10^5^ cells/well) or 10 mm glass bottom dishes (1×10^5^ cells/well), stimulated with the indicated reagents or infected with *E. coli* at a multiplicity of infection (MOI) of 5 (bacteria per macrophage) or *C. albicans* at an MOI of 1. To inhibit the ROS produced by NADPH oxidase, macrophages were pretreated with 10 µM diphenylene iodonium (DPI) before stimulation or infection. After incubation for the indicated times, macrophages were either fixed or observed under live cell conditions. For DNA detection, 5 µg/ml Hoechst 33342 (Sigma-Aldrich, St. Louis, MO, USA), 5 µM SYTOX Green (Invitrogen) or 5 µg/ml PI (Invitrogen) was used. To observe the entrapment of microbes by METs-LS, GFP-labeled *E. coli* was observed directly, and *C. albicans* was stained extensively with calcofluor white (Sigma-Aldrich). For quantification of METs-LS production, the total number of macrophages and the number of macrophages releasing METs-LS per field of view were counted in 5 individual images per sample. At least 400 cells in total were examined for each sample. Macrophages releasing DNA to form extracellular structures were considered as producing METs-LS, and each METs-LS structure was considered been released by one macrophage. For immunofluorescence staining, *C. albicans* infected cells were fixed with cold methanol (to detect histone) or 4% PFA (to detect other proteins) for 15 min, permeabilized with 0.5% Triton X-100 for 2 min and blocked in 1% BSA/PBS overnight. The samples were subsequently incubated with rabbit anti-histone H_2_A (Sigma-Aldrich, 1∶50), rabbit anti-myeloperoxidase (MPO) (Santa Cruz, USA, 1∶50) or goat anti-lysozyme (Santa Cruz, 1∶50) antibodies for 120 min at room temperature, followed by incubation with TRICT-labeled goat anti-rabbit IgG (Sigma-Aldrich, 1∶32) or Cy3 labeled rabbit anti-goat IgG (Boster, Wuhan, China, 1∶50) for 120 min. The samples were then counterstained with 5 µg/ml Hoechst 33342 for 2 min and mounted using a drop of SlowFade Gold antifade reagent (Invitrogen), and examined using fluorescence microscope (Olympus BX53) or a laser scanning confocal microscope (Olympus FluoView FV1000).

### Scanning Electron Microscopy

J774A.1 macrophages were plated onto glass cover slides and infected with *E. coli* or *C. albicans* as in the fluorescence microscopy experiments. After 180 min of incubation, the samples were fixed with 2.5% glutaraldehyde, washed with water and post-fixed with 0.5% osmium tetroxide, followed by 1% tannic acid. Subsequently, the samples were dehydrated with a graded ethanol series (30%, 50%, 70%, 80%, 90%, 100%), critical point dried with liquid CO_2_ and coated with 5-nm platinum. The samples were examined using a scanning electron microscope (Hitachi S-3400N, Japan).

### Microbial killing Assays

A plate killing assay was performed as previously described [32], with slight modifications. Briefly, J774A.1 macrophages or murine peritoneal macrophages were suspended in serum-free DMEM and plated into 24-well plastic plates at a concentration of 1×10^6^ cells/well. Cytochalasin D (Sigma-Aldrich, 10 µg/ml), Protease-free DNase I (Fermentas, USA, 50 U/ml) or both were added to the cell cultures at 20 min before infection with microbes. *E. coli* was added to the cultured cells at a MOI of 5 and *C. albicans* was added at a MOI of 1. The samples were centrifuged at 300 *g* for 5 min and incubated at 37°C with 5% CO_2_ for the indicated times. At 5 min before the end of the incubation, DNase I was added to the wells that did not contain it to release the trapped microbes from the METs-LS. The 24-well plate was subsequently agitated using a micro-plate mixer adjusted to high speed switch. The aliquots were collected, sequentially diluted in cold dilution buffer (0.9% NaCl, pH = 11), and plated on LB agar plates (*E. coli*) or YPD agar plates (*C. albicans*) to determine the CFUs. In addition, the residual medium in the 24-well plate was aspirated, and the wells were washed extensively with 500 µl of cold H_2_O (pH = 11). This washing procedure lyses macrophages and recovers the phagocytosed microbes as well as the microbes remaining in the wells. The CFU of the recovered microbes in washing buffer was also determined as described above. The combination of the two recovered CFUs reflected the quantities of surviving microbes in the wells.

The microbial viability was also determined *in situ* using propidium iodide (PI) staining. PI is a membrane impermeable nucleic acid dye that is commonly used to identify dead cells [33], [34]. Macrophages were seeded onto glass cover slides and infected with GFP-labeled *E. coli* or *C. albicans*. After an incubation of 120 min, the samples were stained with 5 µg/ml Hoechst 33342 and 5 µg/ml PI for 15 min; the METs-LS, as well as dead microbes and dead macrophages, were stained red by PI. The viable and dead microbes were identified under microscope with high magnification. Heat-inactivated *C. albican* and streptomycin sulfate inactivated *E. coli* were used as positive PI staining controls. The samples were washed gently with warm PBS for 3 times and observed immediately using fluorescence microscope. The percentage of dead cells in microbial population trapped by METs-LS was calculated by the ratio of dead cells (red) to the total number of trapped microbes, and at least 10 METs-LS fibers were counted per sample done in triplicate.

### PCR

METs-LS were induced by incubating J774A.1 macrophages with *C. albicans* for 180 min as described above. Extracellular DNA in the supernatant of this culture or control group was purified using classic phenol/chloroform extraction and directly used as templates for PCR. Two mitochondria-specific primers (Atp6, Forward: 5′-AGGATTCCCAATCGTTGTAGCC-3′, reverse: 5′-CCTTTTGGTGTGTGGATTAGCA-3′. Nds1, Forward: 5′-TCACTATTCGGAGCTTTACGAGC-3′, reverse: 5′-CATATTATGGCTATGGGTCAGGC-3′) and two nuclei-specific primers (Gapdh, Forward: 5′-AAGGGCATCTTGGGCTACAC-3′, reverse: 5′-TAGGGCCTCTCTTGCTCAGT-3′. Actinβ, Forward: 5′-CTGTCGAGTCGCGTCCA-3′, reverse: 5′-CGCAGCGATATCGTCATCCAT-3′) were used to determine the origin of extracellular DNA by PCR reaction. The cycling parameters were as follows: 95°C for 1 min, 55°C for 30 s, 72°C for 1 min, 32 cycles. The PCR products were separated on 2% agarose gel and observed under UV light.

### Fluorescence *in situ* Hybridization Analysis

The DNA fragments for making hybridization probes were amplified from the total cellular DNA of J774A.1 cells as follows: the mitochondria-specific fragments (3165 bp) used forward primer 5′-TCCCACTGTACACCACCACATCA-3′ and reverse primer 5′-TGGGGATTGAGCGTAGAATGGCGT-3′. The nuclei-specific fragments (5049 bp), used forward primer 5′-ATGCCGCCATGGCTGTCACT-3′ and reverse primer 5′-ACAGCGCGGTACACAAGCCA-3′. Both fragments were nick-translated and labeled with red fluorescent dye Alexa Fluor 594 using the FISH tag DNA kit (Invitrogen) according to the manufacturer’s instructions.

J774A.1 macrophages were seeded onto on glass cover slides and stimulated with *C. albicans* for 180 min as described above. The cells were fixed with 4% PFA, subjected to dehydration/rehydration in serial graded ethanol solutions and treated with DNase-free RNase A (Fermentas). The hybridizations were performed overnight at 37°C using the FISH tag DNA kit according to the manufacturer’s protocols. The samples were counterstained with Hoechst 33342, mounted with SlowFade Gold antifade reagent and analyzed using fluorescence microscope.

### Reactive Oxygen Species (ROS) Detection

The intracellular ROS production of activated J774A.1 macrophages was detected using 2′, 7 dichlorofluorescein diacetate (DCFH-DA) (Sigma), which is converted to highly fluorescent dichlorofluorescein (DCF) in the presence of intracellular ROS. Macrophages were seeded onto glass cover slides in 24-well plates (2×10^5^ cells/well) or plated in 96-wells plate with black frames (1×10^5^ cells/well) and infected with *E. coli* or *C. albicans*, or stimulated with 100 nM PMA. DCFH-DA was added to the cultures at a final concentration of 5 µM. The samples on cover slides were incubated at 37°C and 5% CO_2_ for 60 min. The DNA was stained with Hoechst 33342 for 5 min and the samples were observed under fluorescence microscopy. The samples in the plates were incubated for the indicated times and washed twice with PBS. The fluorescence intensity was then measured on a fluorescence plate reader (Tecan infinite F200) at 450 nm excitation and 530 nm emission.

### Lactate Dehydrogenase (LDH) Activity Detection

J774A.1 macrophages were seeded onto 96-well cell culture plates at a concentration of 1×10^5^ cells/well. *E. coli* or *C. albicans* were added to the wells and incubated for the indicated time points. The plates were centrifuged at 300 *g* for 5 min to collect the supernatants. The LDH activity in supernatants was determined using an LDH Cytotoxicity Assay Kit (Roche Applied Science, Indianapolis, USA), and the control group and positive lysis group were determined according to the manufacturer’s protocol after an incubation of 180 min.

### Statistical Analysis

All results were expressed the as the means ± SD. The group means were compared using one-way ANOVA, and Student’s t-test was used to determine the significance of differences. P values of 0.05 or less were considered statistically significant.

## Results

### *E. coli* and *C. albicans* Induce and are Trapped by Macrophage Extracellular Structures

**Figure 1:**
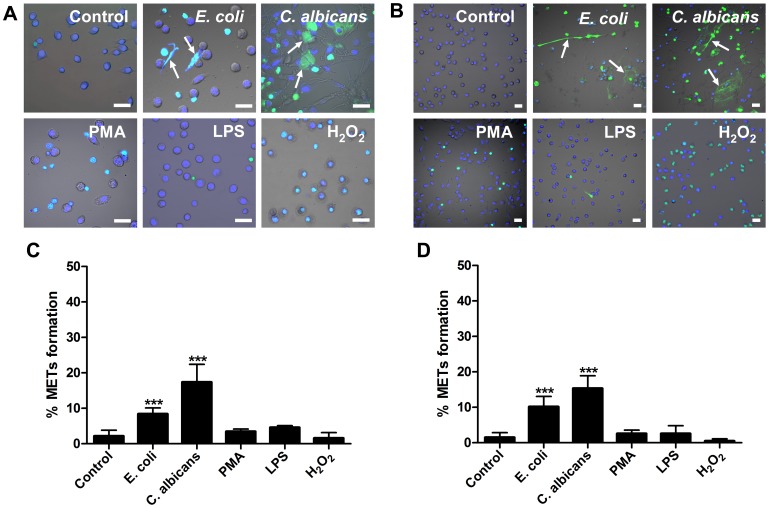
*E. coli* and *C. albicans* induce METs-LS formation in murine macrophage cells. **A–B:** Murine J774A.1 macrophages (A) or peritoneal macrophages (B) were cultured in serum-free DMEM medium and stimulated with *E. coli* (MOI 5), *C. albicans* (MOI 1), PMA (10 µM), LPS (50 µg/ml), hydrogen peroxide (10 mM) or vehicle control at 37°C for 180 min. Hoechst 33342 (Blue) and SYTOX Green (Green) was added to stain the DNA. Fluorescence staining images merged with differential interference contrast are shown. The arrows indicate METs-LS released by macrophages in response to *E. coli* or *C. albicans*. Scale Bars: 20 µm. **C–D:** The quantification of METs-LS positive cells in murine J774A.1 macrophages (C) or peritoneal macrophages (D) stimulated with *E. coli*, *C. albicans*, LPS, PMA or hydrogen peroxide is shown as the means ± SD (n = 5). ***P<0.001 compared with control group by two tailed Student’s t-test, respectively. These experiments were repeated independently 3 times with similar results.

*E. coli* or *C. albicans* were co-incubated with murine macrophage cell line J774A.1 or peritoneal macrophages for 180 min. Fluorescence imaging analysis revealed that in both cell types, both pathogens induced the release of extracellular fibers or reticulation structures, detected with the non-cell-permeable DNA dye SYTOX Green (Green) and cell-permeable DNA dye Hoechst 33342 (Blue) (Figure 1*A–B*). This indicated that DNA is an important component of these matrixes, which is a typical characteristic of ETs. We defined these matrixes as macrophage extracellular traps-like structures (METs-LS). Macrophages without microbial stimulation produce relatively few of METs-LS (Figure 1*A–B*). The yeast-form of *C. albicans* was also able to induce the formation of METs-LS under the 30°C culture condition (Figure S1 *A–B*).

We next examined the effects of chemical regents such as LPS, PMA and hydrogen peroxide on the induction of METs-LS formation. None of these regents stimulated METs-LS formation in murine J774A.1 or peritoneal macrophages (Figure 1*A–B*). PMA and hydrogen peroxide treatments only led to death of a portion of macrophages, which did not release extracellular DNA. Further, macrophages pretreated with IFN-γ (100 U/ml) or M-CSF (25 ng/ml) were not responsive to stimulation by these compounds in the formation of METs-LS (data not shown).

Quantification of METs-LS formation revealed that co-culture of murine macrophages with microbes significantly increased the amount of cells producing METs-LS, while the portions of cells undergoing METs-LS formation among the chemical regent-treatment and control groups were not significantly different (Figure 1*C–D* *;* Figure S1 *C–D*). Moreover, the production of METs-LS is dependent on the type of microbes used. *C. albicans* hyphae induce 17% of J774A.1 macrophages to form METs-LS after 180 min incubation, which was higher than that of *E. coli* (8%) or the yeast-form of *C. albicans* (10%). Similar phenomenon was also observed in peritoneal macrophages, in which *C. albicans* hyphae induces 15% macrophages, *E. coli* and the yeast-form of *C. albicans* induce 10% and 8.5% macrophages to form METs-LS, respectively.

**Figure 2:**
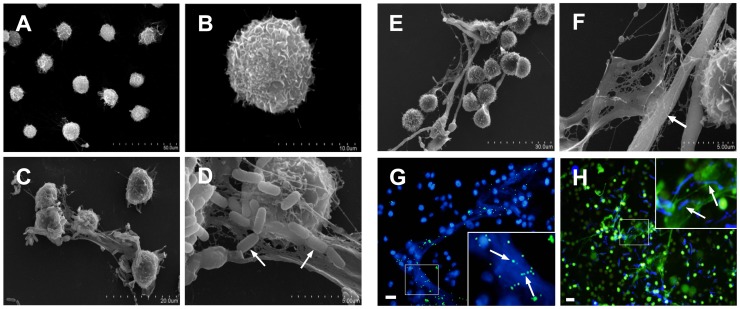
Scanning electron microscopy (SEM) and fluorescence micrographs of microbes trapped by METs-LS. J774A.1 macrophages were seeded onto glass cover slips and incubated with *E. coli* or *C. albicans* for 180 min. **A–B:** SEM images of uninfected J774A.1 cells. **C–D:** SEM images of METs-LS induced by *E. coli*. The white arrows indicate the bacteria adhered to the surface of METs-LS. **E–F:** SEM images of METs-LS induced by *C. albicans*. The white arrows indicate the fungus trapped in the METs-LS. **G:** Fluorescence photograph of DNA (Blue) and FITC-labeled *E. coli* (Green). **H:** Fluorescence photograph of DNA (Green) and calcofluor white-stained *C. albicans* (Blue). Both microbes co-localized with METs-LS. In (G) and (H), samples were washed twice with PBS to remove unbounded microbes before analysis. These experiments were repeated independently 3 times with similar results. Scale Bars: 20 µm.

Scanning electron microscopy was used to observe the ultrastructure of METs-LS. Rarely extracellular structures were observed in un-stimulated J774A.1 macrophages (Figure 2*A*–*B*). J774A.1 macrophages challenged by *E. coli* or *C. albicans* formed METs-LS; and the microbes were observed to bind to the surface of these structures (Figure 2*C*–*F*). The METs-LS were released from the macrophage membrane, and the morphology of these cells remained unchanged (Figure 2*C*–*F*). Fluorescence images also showed that the microbes co-localized with the METs-LS, indicating that the METs-LS entrapped microbes (Figure 2*G*–*H*).

### METs-LS Show Limited Killing Effects on *E. coli* but not *C. albicans*

**Figure 3:**
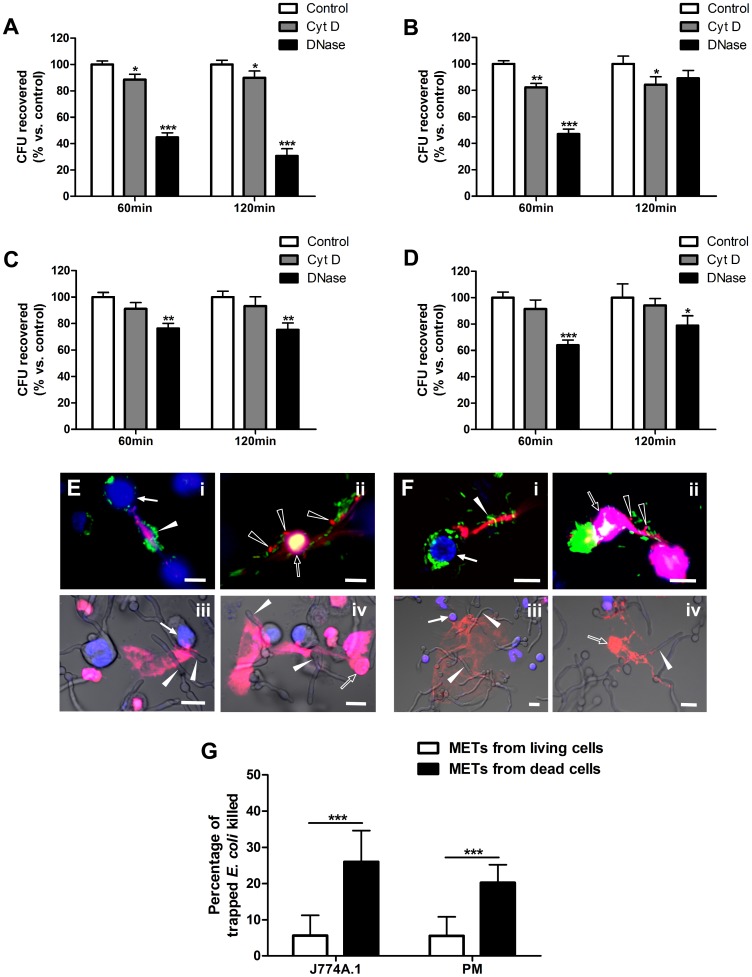
METs-LS show limited killing effect on *E. coli* but not on *C. albicans*. J774A.1 macrophages or murine peritoneal macrophages were infected with *E. coli* or *C. albicans* and incubated for the indicated time points. **A–B:** The survival rates of *E. coli* (A) and *C. albicans* (B) incubated with J774A.1 macrophages. **C–D:** The survival rates of *E. coli* (C) and *C. albicans* (D) incubated with peritoneal macrophages. Experiments were performed 3 times with similar results, and a representative experiment is shown as the means ± SD (n = 3). *P<0.05, **P<0.01 and ***P<0.001 compared with the control groups by two tailed Student’s t-test, respectively. **E–F:** PI staining was performed to determine the dead macrophages and dead microbes trapped in the METs-LS formed by J774A.1 cells (E) and peritoneal macrophages (F) after 120 min co-incubation. Macrophages were labeled with Hoechst 33342 (Blue). METs-LS, dead macrophages and dead microbes were stained positive by PI (Red). The solid and hollow arrowheads indicate viable and dead microbes in *E. coli* (i-ii) or *C. albicans* (iii-iv) infected groups, respectively. Only a portion of GFP- *E. coli* trapped by METs-LS released from dead macrophages was killed. Scale Bars: 10 µm. **G:** The quantification of dead *E. coli* trapped in METs-LS released from viable and dead J774A.1 macrophages and peritoneal macrophages, result is shown as the means ± SD (n = 10), ***P<0.001 compared with control group by two tailed Student’s t-test. These experiments were repeated independently 3 times with similar results.

Previous studies have shown that extracellular traps from neutrophils, eosinophils and mast cells kill various microbes [5], [6], [7]. Here, we determined the microbicidal activity of murine METs-LS by plate assay. Macrophages were treated with cytochalasin D (which did not affect METs-LS formation [29]) to block phagocytosis or DNase I to degrade METs-LS. Samples pre-treated with both cytochalasin D and DNase I were set as the 100% survival control group [35]. After incubation with *E. coli* or *C. albicans* for the indicated durations, the surviving microbes in the supernatant of each group were calculated. METs-LS-mediated killing was represented in the cytochalasin D-treated group and phagocytic killing was represented in the DNase I-treated group. Compared to the control group, J774A.1 METs-LS killed 11% and phagocytosis killed 55% of the *E. coli* after 60 min incubation. When the incubation was extended to 120 min, a similar result was observed (Figure 3*A*). The METs-LS of J774A.1 macrophages also showed limited anti-Candida activity in this assay. 60 min incubation resulted in 18% reduction of *C. albican*s compared to the control group. This killing efficiency was not affected by longer incubation time up to 120 min (Figure 3*B*). The phagocytosis of J774A.1 macrophages was able to reduce 53% *C. albicans* after the 60 min incubation. Unexpectedly, this phagocytic killing was significantly reduced after the 120 min incubation (Figure 3*B*). Such increase may result from the growth of engulfed *C. albicans* in macrophages.

The antimicrobial ability of murine peritoneal macrophage-released METs-LS was also examined by the plate assay. The results revealed that the amounts of both *E. coli* and *C. albicans* recovered from the cytochalasin D-treated groups were insignificantly lower than those of the control groups, suggesting an ineffective microbicidal activity of peritoneal METs-LS. Whereas phagocytosis eliminated 24% of the *E. coli* and 36% of the *C. albicans* after incubation for 60 min, and approximate 20% of both microbes after incubation for 120 min (Figure 3*C*–*D*).

PI staining was performed to determine the viability of the microbes trapped in the METs-LS, in which dead microbes were stained red (Figure S2). We found that a portion of microbes induced METs-LS were released by dying macrophages. No METs-LS was observed in necrotic macrophages (Figure S3), which imply this METs-LS associated cell death was different from necrosis. Meanwhile, METs-LS were also released from viable macrophages. It was notable that almost all the *E. coli* cells trapped by the METs-LS released from viable macrophages showed positive GFP-fluoresence and were stained negative by PI (Figure 3*E* *_i_* and *F_i_)*, which indicated these bacteria were still alive. In contrast, a small portion of *E. coli* trapped by METs-LS released from dying macrophages were stained red, suggesting that killing occurred (Figure 3*E* *_ii_* and *F_ii_*). To quantify the bactericidal activity of these two METs-LS, we counted the mortality of *E. coli* trapped by these METs-LS, respectively. We noticed a small portion of METs-LS were associated with several macrophages which contain both viable and dead cells, thus made it difficult to judge whether they were released from a viable or dead macrophage. These METs-LS were excluded in this experiment. We found the METs-LS released from dead J774A.1 macrophages (nuclei stained red) show significant higher bactericidal activity than that from viable macrophages (Figure 3*G*). Similar results were also observed in peritoneal macrophages (Figure 3*G*). However, none of the trapped *C. albicans* was observed to be dead on both types of METs-LS (Figure 3*E* *_iii-iv_* and *F_iii-iv_*). These results indicated that METs-LS released from macrophages that had undergone cell death, but not macrophages which stayed alive, possess the ability to kill *E. coli*. *C. albicans* showed resistance to METs-LS mediated killing, and the reduction of *C. albicans* in plate assay implied that their proliferation may be partly inhibited by METs-LS.

### Macrophages Release Both Mitochondrial and Nuclear DNA to form METs-LS

**Figure 4:**
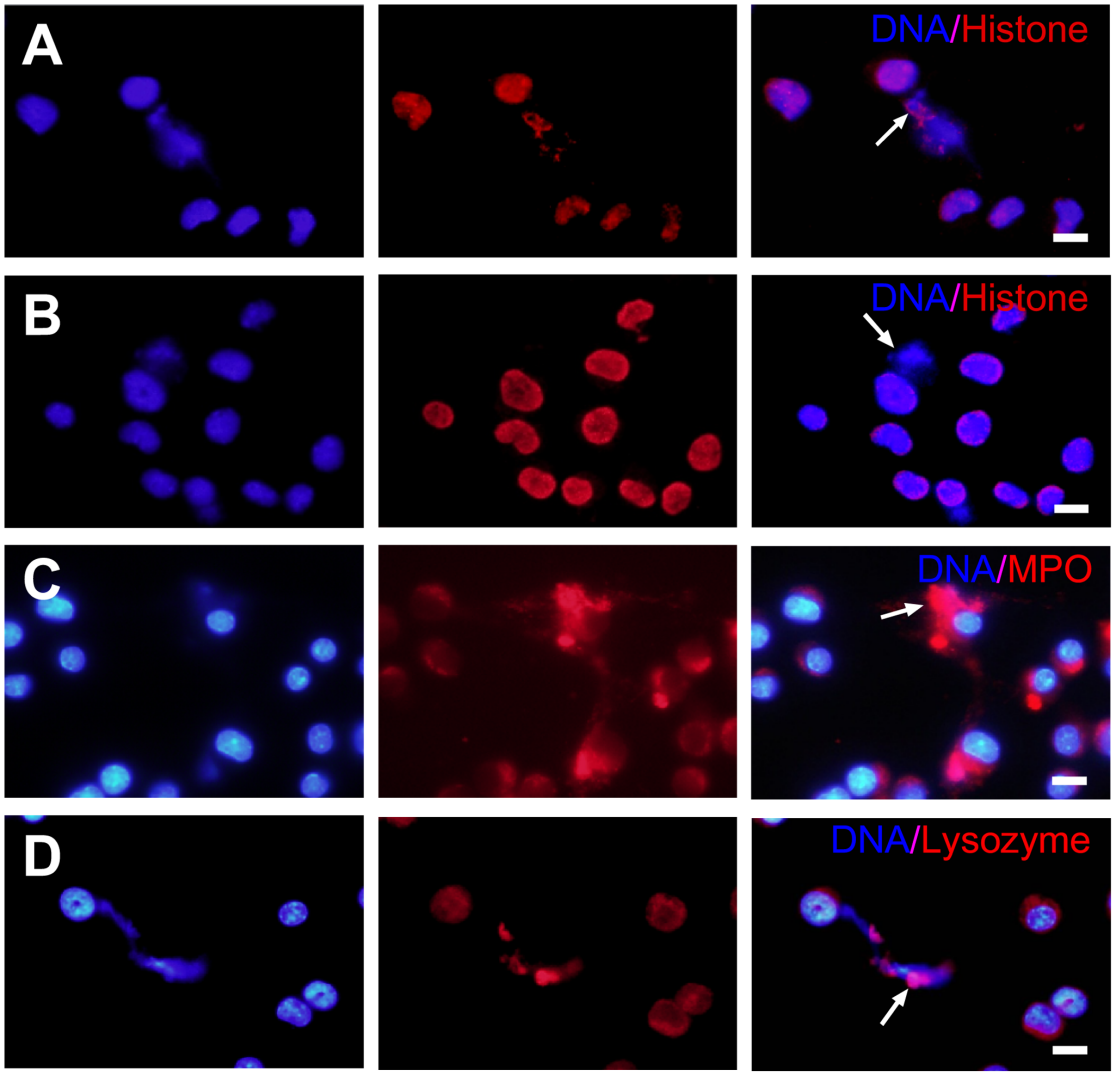
METs-LS contain histone, MPO and lysozyme. METs-LS were induced by incubation murine J774A.1 macrophages with *C. albicans* at a MOI of 1 for 180 min. The cells were fixed, permeabilized, blocked and incubated with anti-histone (**A–B**), anti-MPO (**C**) or anti-lysozyme (**D**) primary antibodies, followed by staining with TRICT or Cy3-labeled secondary antibodies (Red). The DNA was stained with Hoechst 33342 (Blue). The arrows indicate METs-LS structures. The result shown in Panel B reveals that some METs-LS do not contain histone. Scale Bars: 10 µm. These experiments were repeated 3 independent times with similar results.

To examine the composition of METs-LS, we conducted immunofluorescence staining on *C. albicans* stimulated J774A.1 macrophages. In addition to the DNA backbone, we found that some microbicidal proteins, including histone [36], MPO and lysozyme, were co-localized with METs-LS (Figure 4*A, C, D*). Notably, we found that not all observed METs-LS were positively stained for histone (Figure 4*B*), indicating more than one composition existed in the microbe induced murine METs-LS. Because histones were commonly associated with nuclear DNA, we proposed that the DNA backbone of METs-LS is originated from either nuclear or mitochondrial.

**Figure 5:**
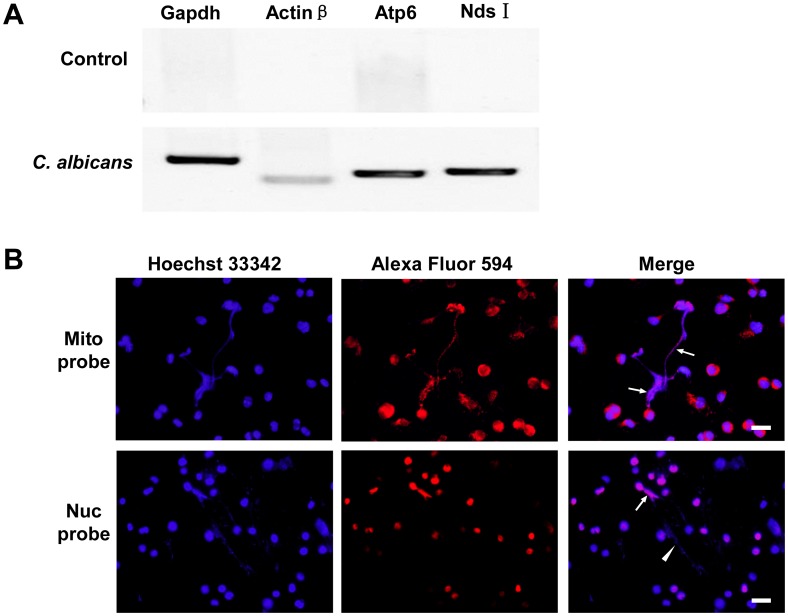
Macrophages release both mitochondrial and nucleus DNA to form METs-LS. **Panel A:** Two mitochondrial genes (*Atp6*, *Nds1*) and two nuclear genes (*Actinβ*, *Gapdh*) were amplified from different DNA template by PCR. Both mitochondrial and nuclear genes were detectable in the supernatants of *C. albicans* infected J774A.1 macrophages but not in control group. **B:** Fluorescence *in situ* hybridization analysis of *C. albicans*-induced J774A.1 METs-LS. DNA was labeled with Hoechst 33342 (Blue). The arrows indicate the positive signals in the Alexa Fluor 594 (Red)-labeled nucleus (Nuc) or the mitochondria (Mit) specific DNA probe in macrophage extracellular DNA. The arrowhead indicates that some extracellular DNA did not stain for the nuclear DNA probe. Scale Bars: 20 µm. These experiments were repeated independently 3 times with similar results.

To determine the origin of the extracellular DNA, PCR and fluorescence *in situ* hybridization experiments were performed. We amplified both mitochondrial (*Atp6* and *Nds1*) and nuclear (*Actinβ* and *Gapdh*) genes from the supernatant of *C. albicans*-stimulated macrophages, with no amplification in the control group (Figure 5*A*). Sequencing of these fragments indicated all of them were specifically amplified. Fluorescence *in situ* hybridization with mitochondrial and nuclear DNA probes confirmed the PCR results. The mitochondria-specific probe showed cytoplasm localization and nuclei-specific probe showed nucleus localization in macrophages which didn’t release METs-LS (Figure 5*B*). Positive signals from mitochondria- and nuclei-specific probes were both observed after hybridization with METs-LS (Figure 5*B*). Notably, only a portion of the METs-LS showed positive signals with the nucleus-specific probe (Figure 5*B*), whereas the mitochondria-specific probe reacted with all observed METs-LS. These observations indicate that the DNA backbone of METs-LS originated from the mitochondrion or both the mitochondria and the nucleus.

### METs-LS Formation is Independent of ROS Produced by NADPH Oxidase

**Figure 6:**
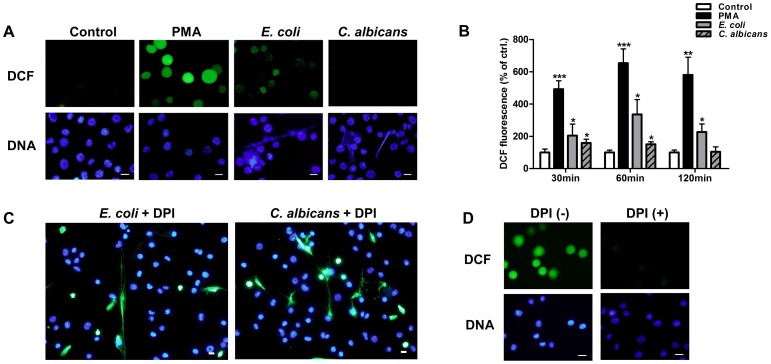
Formation of METs-LS is independent of ROS produced by NADPH oxidase. **A:** Fluorescence microscope determination of intracellular ROS production in PMA (positive control), *E. coli-* and *C. albicans*-stimulated J774A.1 macrophages using DCF ROS probe (Green). DNA was stained with Hoechst 33342 (Blue). The arrows indicate macrophages releasing METs-LS. **B:** The quantification of ROS production in negative, positive (PMA), *E. coli-* and *C. albicans-*infected macrophage groups. The fluorescence intensity of the ROS probe was measured using a fluorescence plate reader. The data are presented as the means ± SD of three independent experiments. *P<0.05 and ***P<0.001 compared with the medium-only culture control group by ANOVA with Bonferroni’s post-test. **C:** Determination of *E. coli* or *C. albicans* induced J774A.1 METs-LS in the presence of 10 µM DPI. Hoechst 33342 and SYTOX Green were added to assess METs-LS formation. **D:** Determination of intracellular ROS production in PMA stimulated J774A.1 macrophages in the presence or absence of 10 µM DPI. DNA was stained with Hoechst 33342 and intracellular ROS was determined by DCF ROS probe. Scale Bars: 10 µm. These experiments were repeated independently 3 times with similar results.

To investigate the role of ROS production in METs-LS formation, we determined ROS production of J774A.1 macrophages using a DCF fluorescence probe (Green). *E. coli* or *C. albicans* were used to stimulate METs-LS formation, and the METs-LS were stained with Hoechst 33342. Macrophages which released METs-LS were negatively stained with ROS probe, indicating that these cells did not undergo ROS production (Figure 6*A*). Whereas after treating the macrophages with 100 nM PMA (positive control) for 60 min, which did not induce METs-LS formation, most cells were positively stained for DCF (Figure 6*A*). We also determined the level of ROS production using a fluorescence intensity reader. The results showed that PMA and *E. coli* induced significant amounts of ROS production after incubation for 30 min (Figure 6*B*). *C. albicans* only induced low amounts of ROS production in the early phase of infection, which was abolished after 120 min (Figure 6*B*). As *C. albicans* are more effective at inducing METs-LS formation than *E. coli*, the production of ROS may not be a prerequisite for this process.

The NADPH oxidase inhibitor DPI was used to determine whether microbes induced METs-LS formation depended on ROS produced by NADPH oxidase. We found that addition of 10 µM DPI did not impede *E. coli* and *C. albicans* induced METs-LS formation (Figure 6*C*). The inhibition of NADPH oxidase mediated ROS production by DPI at the concentration used in this study was confirmed by using DCF fluorescence probe in PMA stimulated macrophages (Figure 6*D*). These results suggest that J774A.1 macrophages release METs-LS in response to microbes in a NADPH oxidase-independent manner.

### METs-LS Formation is Independent of Cell Lysis

**Figure 7:**
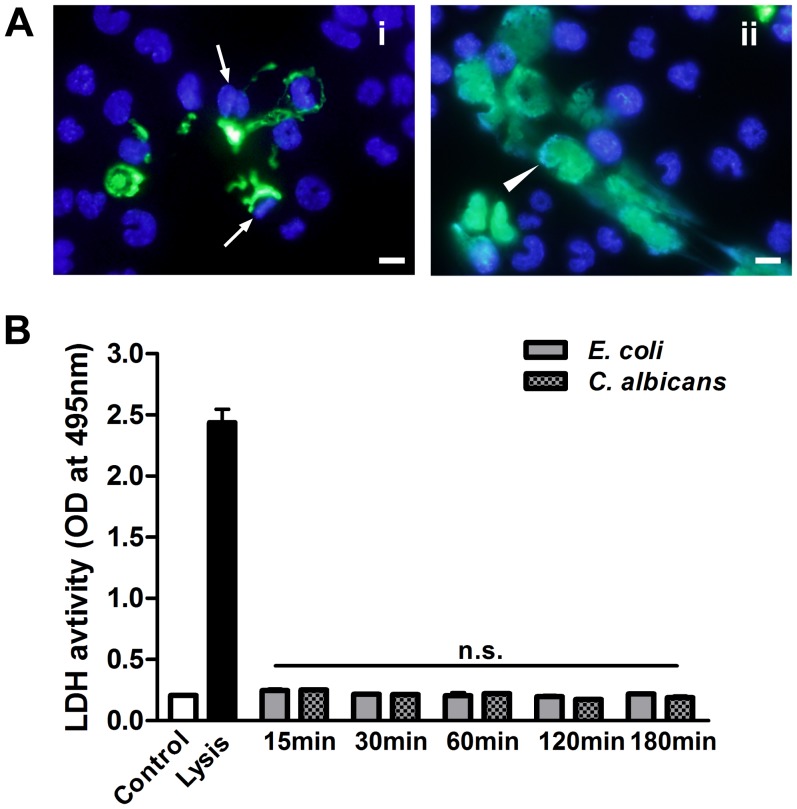
METs-LS-induced cell death is independent of necrosis. **A:** The J774A.1 METs-LS induced by *C. albicans* were stained with SYTOX Green and Hoechst 33342, revealing that the METs-LS are released from either viable (i) or dead (ii) macrophage cells after 120 min incubation. The arrows indicate viable METs-LS formation cells with unchanged nuclei shape, the arrowheads indicate dead MET-LS formation cells with enlarged nuclei. Scale Bars: 10 µm. These experiments were repeated independently 3 times with similar results. **C:** J774A.1 macrophages were infected with *E. coli* or *C. albicans* for 15, 30, 60, 120 and 180 min, respectively. The LDH level in the supernatant of each group was quantified. The data are presented as the means ± SD of three independent experiments.

To determine the viable status of macrophages, the non-cell-permeable DNA dye SYTOX Green was used to stain extracellular DNA and dead cells. We observed that microbes induced METs-LS were released from either viable J774A.1 cells with unchanged nuclei shape (Figure 7*A* *_i_*) or dying J774A.1 cells which exhibited enlarged nuclei (Figure 7*A* *_ii_*). These METs-LS existed together after the stimulation of macrophages with microbes.

The supernatant LDH level (an indicator of membrane permeabilization) assay was performed to determine whether METs-LS releasing by dying cells is associated with membrane damage. J774A.1 macrophages were co-incubated with *E. coli* or *C. albicans* for 15, 30, 60, 120 and 180 min to induce METs-LS formation, and subsequently the LDH activity in the supernatant was determined. Untreated J774A.1 macrophages and cells treated with lysis buffer were used as negative and positive controls, respectively. Although *E. coli* and *C. albicans* induced METs-LS formation and cell death, this process was not concomitant with a detectable increase in the LDH level (Figure 7*B*). This result suggests that the macrophage death in METs-LS formation process did not lead to cell lysis, which was different from necrosis.

## Discussion

Several studies have indicated that the formation of extracellular traps is an effective innate immune mechanism used to combat invading pathogens. However, this mechanism in macrophages is relatively poorly understood. Here, we observed that *E. coli* (MOI 5) and *C. albicans* (MOI 1) induce METs-LS formation in both the murine J774A.1 macrophage cell line and peritoneal macrophages. After infection with these two microbes, only a subset (∼10%–20%) of macrophages formed METs-LS, which was similar to the METs induced by other stimuli [8], [29], [31]. Infection with higher MOI of microbes led to cell lysis of macrophages but did not significantly increase METs-LS formation after 180 min incubation (data not shown). Our results also indicate that METs-LS, similar to ETs produced by other immune cells, are significantly associated with the microbial surface. The molecular mechanism of such association remains unknown, but it is possible that electrostatic interactions between the cationic components of ETs and the anionic surface of microorganisms may be involved [37]. ETs have been proposed to restrict microbes to the site of infection, thus preventing them from systemic spread, a function important for controlling invasive infections, particularly those caused by fungal pathogens [14], [18], [38]. The fact that macrophages are present in various tissues and often are the first innate immune cells to encounter invading pathogens [39], [40] further highlight the importance of METs-LS in controlling infections. These structures may delay the spread of pathogens after they pass through the epithelial barrier, thereby providing time to recruit other effector cells, such as neutrophils, to the infection site for the elimination of the pathogens. We also found the *C. albicans* hyphae induce more METs-LS than its yeast-form and *E. coli*, which was similar to the observation described for NETs [9]. At least two reasons can contribute to this phenomenon: first, because of its linear shape, *C. albicans* hyphae cannot be efficiently engulfed by macrophages, thus allowing longer extracellular stimulation for METs-LS formation; second, the hyphae form is more relevant to the invasion and spread phase of *C. albicans* infection [41], which might be more efficient to induce METs–LS formation.

Several chemical stimuli, including PMA, LPS and hydrogen peroxide, are effective inducers for ETs [5], [7], [42]. However, the ability of LPS to induce ETs formation remains controversial. Some studies reported that LPS induced the release of ETs from neutrophils or eosinophils only after pre-treating the cells with certain cytokines [6], [22] or in combination with platelets under flow conditions [43]. In our experiments, we unexpectedly observed that murine macrophages did not form METs-LS in response to stimulation by PMA, LPS or hydrogen peroxide, even after priming these cells with cytokines such as IFN-γ or M-CSF. A previous study indicated that in bovine macrophages higher concentrations of PMA and glucose oxidase (an H_2_O_2_-producing enzyme) are required to stimulate METs-LS formation than those needed for the induction of NETs [29]. In contrast, we found that stimulation of murine macrophages with >10 µM of PMA or >10 mM of H_2_O_2_ resulted in only death of the cells, but not the formation of METs-LS. Although it was reported that neutrophil produce significant amount of NETs after stimulated with 25 nM PMA or 10 mM H_2_O_2_ [5], [44], a study on NETosis indicated H_2_O_2_ only induced apoptotic or necrotic cell death of neutrophil, but not NETosis [23]. As challenge by microbes is a more effective METs-LS stimulus than chemicals, we propose that physical stimulation following the activation of cell receptors is essential to initiate this METs-LS formation process.

A recently published report indicates that NETs entrap, but do not kill the microbes [32]; the microbicidal ability of NETs may have been overestimated in previous studies, probably due to the limitation of methods for killing analysis [32]. By the modified plate assay protocol and *in situ* PI staining, our results revealed that METs-LS kill only a portion of entrapped *E. coli*, but not *C. albicans*. These results differ from the fact that ETs contain various antimicrobial agents. It is possible that the antimicrobial activity of these components has been compromised in the extracellular environment. In agreement with this notion, the antibacterial peptide CRAMP associated with NETs exhibited significantly reduced antimicrobial activity against *S. aureus* [45]. The loss of antimicrobial activity of by CRAMP may result from its association with DNA, whose anionic property might reduce access of the peptide to the microbial cell surface. Similarly, the cationic agents in METs-LS may also have reduced antimicrobial activities, leading to poor killing potentials of entrapped microbes. Interestingly, we observed that the killing of bacteria primarily occurred in the METs-LS which were released by dying macrophages. These type of METs-LS contain nuclear histones [8], which are very effective microbicidal proteins [36] and may contribute to the bactericidal activity of the METs-LS.

Granulocyte ETs structures contain an extracellular DNA scaffold and various antimicrobial proteins and peptides [5], [14], [46]. Similarly, we found that murine J774A.1 METs-LS consisting of histones, MPO and lysozymes. The distribution of histones in METs-LS reveals that the microbe-induced METs-LS may possess more than one composition. We also detected both nuclear and mitochondrial DNA in J774A.1 METs-LS, suggesting that macrophages form these structures in response to microbes through both the “nuclear extrusion” [21] and the “mitochondrial catapult” [6] mechanisms. The DNA in ETs released by microbes-induced neutrophils and mast cells was generally thought to be originated from the nucleus [5], [7], [16]. Because the release of mitochondrial DNA to the extracellular milieu is a process more rapid than the release of nuclear DNA [6], [22], we speculate that this pattern contributes to the initial capture of microbes by macrophage after recognition. The signaling pathways that govern the mechanisms of the formation of these two METs-LS await future investigation.

The formation of ET in neutrophils, eosinophils and mast cells upon stimulation depends on the generation of ROS through the activation of the NADPH oxidase [6], [7], [21]. Some microbes, such as *Staphylococcus aureus* [16], *Leishmania donovani* [25] and *C. albicans* [26] induce NETs formation in an ROS-independent manner, which is consistent with our observation that METs-LS formation in response to *E. coli* and *C. albicans* appears independent of ROS production. Similarly, the NADPH oxidase inhibitor DPI was unable to abolish METs-LS production. High concentration of DPI have been shown to inhibit bovine METs-LS formation [29]. Because this compound is toxic to both the host cells and microbes [47], [48], [49], such inhibition may be caused by its non-specific toxicity. Nevertheless, much remains to be done in the elucidation of the molecular mechanisms underlying ROS-independent ETs formation.

The NETs-induced cell death was recently designated as a novel form of cell death - NETosis [46]. This type of cell death occurs by a series of events, including chromatin homogenization, nuclear envelope formation, granule membranes disintegration, NETs component mixing and cell membrane breakage [21]. However, whether the macrophages cell death upon METs-LS formation possesses similar mechanism to NETosis is still unclear. Lack of specific markers (such as citrullinated histone in NETosis) for “METosis” also made it difficult to distinguish this type of cell death from necrosis in quantitative assays. It has been reported that unlike necrosis, several stimuli induced NETosis do not promote the release of LDH, which indicates that NETosis do not accompany cell lysis [5], [16], [50], [51]. We found some macrophages undergo cell death during METs-LS formation (Figure 3*E*–*F*), and there was also no detectable LDH activity increase (Figure 7*B*). As METs-LS released from viable macrophages appeared deficient in killing microbes, we propose that the cell death associated with METs-LS production is a “suicide attack”, in which activated macrophages are induced to release both nuclear and cytoplasmic microbicidal agents for strengthening the microbicidal activity of METs-LS.

In conclusion, our study demonstrates that compared to NETs, the murine J774A.1 METs-LS possess some unique characteristics in their formation. Instead of killing the invading microbes, the primary function of METs-LS likely is to ensnare invading microbes to prevent their dissemination. We speculate this phagocytosis-independent antimicrobial mechanism serves as a “supplementary” strategy in innate defense when phagocytosis failed to eliminate invaded pathogens. In addition, METs-LS may also participate in mediating certain cytokines release as reported in NETs [52]. Meanwhile, accumulation of METs-LS is potentially harmful to host, because NETs has been proved to participate in initialing or propagating some autoimmune diseases such as systemic lupus erythematosus and small vessel vasculitis [53], [54]. Why only a small portion of macrophages form METs-LS in response to microbes infection is still unclear. As viable macrophages play essential roles in driving adaptive and innate immune through antigen presentation or cytokines and chemokines secretion, this pattern is more likely to be a “self-regulation” because excessive METs-LS formation may damage the ordinary functions of macrophages.

## Supporting Information

Figure S1 ***C. albicans*** **yeast-form induces murine METs-LS formation. A-B:** Murine J774A.1 macrophages (A) or peritoneal macrophages (B) were infected with *C. albicans* (MOI 1∶1) or a vehicle control and cultured at 30°C for 180 min. Hoechst 33342 (Blue) and SYTOX Green (Green) were added to label the nucleus and extracellular DNA. The arrows indicate macrophages releasing METs-LS in response to the *C. albicans* yeast-form. Scale Bars: 20 µm. **C–D:** The quantification of METs-LS-positive cells in murine J774A.1 macrophages (C) or peritoneal macrophages (D) stimulated with the *C. albicans* yeast-form, the result is shown as the means ± SD (n = 5). ***P<0.001 compared with the control group by two tailed Student’s t-test, respectively. The experiments were repeated independently 3 times with similar results.
(TIF)

Figure S2 **Dead** ***E. coli*** **and** ***C. albicans*** **are stained red by PI.** *E. coli* treated with 200 µg/ml streptomycin sulfate for 2 h **(A)** and *C. albicans* treated at 100°C for 15 min **(B)** were used as positive control for PI staining. Scale Bars: 10 µm.
(TIF)

Figure S3 **Necrotic macrophages do not form METs-LS.** PI staining of necrotic J774A.1 macrophages (A) and necrotic peritoneal macrophages (B) induced by hydrogen peroxide (100 µM, 3 h). Scale Bars: 20 µm.
(TIF)

## References

[1] MacMicking J, Xie Q-W, Nathan C (1997) Nitric oxide and macrophage function Annual Review of Immunology. 15: 323–350. 10.1146/annurev.immunol.15.1.323 9143691

[2] Plowden J, Renshaw-Hoelscher M, Engleman C, Katz J, Sambhara S (2004) Innate immunity in aging: impact on macrophage function. Aging Cell 3: 161–167. 15268749 10.1111/j.1474-9728.2004.00102.x

[3] Cutler JE (1991) Putative Virulence Factors of *Candida albicans* . Annual Review of Microbiology 45: 187–218. 10.1146/annurev.mi.45.100191.001155 1741614

[4] Benoit M, Desnues B, Mege J-L (2008) Macrophage Polarization in Bacterial Infections. The Journal of Immunology 181: 3733–3739. 18768823 10.4049/jimmunol.181.6.3733

[5] Brinkmann V, Reichard U, Goosmann C, Fauler B, Uhlemann Y, et al (2004) Neutrophil Extracellular Traps Kill Bacteria. Science 303: 1532–1535. 15001782 10.1126/science.1092385

[6] Yousefi S, Gold JA, Andina N, Lee JJ, Kelly AM, et al (2008) Catapult-like release of mitochondrial DNA by eosinophils contributes to antibacterial defense. Nat Med 14: 949–953. 18690244 10.1038/nm.1855

[7] von Köckritz-Blickwede M, Goldmann O, Thulin P, Heinemann K, Norrby-Teglund A, et al (2008) Phagocytosis-independent antimicrobial activity of mast cells by means of extracellular trap formation. Blood 111: 3070–3080. 18182576 10.1182/blood-2007-07-104018

[8] Chow OA, von Köckritz-Blickwede M, Bright AT, Hensler ME, Zinkernagel AS, et al (2010) Statins Enhance Formation of Phagocyte Extracellular Traps. Cell Host & Microbe 8: 445–454. 21075355 10.1016/j.chom.2010.10.005 PMC3008410

[9] Ermert D, Urban CF, Laube B, Goosmann C, Zychlinsky A, et al (2009) Mouse Neutrophil Extracellular Traps in Microbial Infections. Journal of Innate Immunity 1: 181–193. 20375576 10.1159/000205281 PMC6951040

[10] Lippolis JD, Reinhardt TA, Goff JP, Horst RL (2006) Neutrophil extracellular trap formation by bovine neutrophils is not inhibited by milk. Veterinary Immunology and Immunopathology 113: 248–255. 16806491 10.1016/j.vetimm.2006.05.004

[11] Alghamdi AS, Foster DN (2005) Seminal DNase Frees Spermatozoa Entangled in Neutrophil Extracellular Traps. Biology of Reproduction 73: 1174–1181. 16107606 10.1095/biolreprod.105.045666

[12] Palić D, Ostojić J, Andreasen CB, Roth JA (2007) Fish cast NETs: Neutrophil extracellular traps are released from fish neutrophils. Developmental & Comparative Immunology 31: 805–816. 17222907 10.1016/j.dci.2006.11.010

[13] Kessenbrock K, Krumbholz M, Schonermarck U, Back W, Gross WL, et al (2009) Netting neutrophils in autoimmune small-vessel vasculitis. Nat Med 15: 623–625. 19448636 10.1038/nm.1959 PMC2760083

[14] Urban CF, Ermert D, Schmid M, Abu-Abed U, Goosmann C, et al (2009) Neutrophil Extracellular Traps Contain Calprotectin, a Cytosolic Protein Complex Involved in Host Defense against *Candida albicans* . PLoS Pathog 5: e1000639. 19876394 10.1371/journal.ppat.1000639 PMC2763347

[15] Papayannopoulos V, Zychlinsky A (2009) NETs: a new strategy for using old weapons. Trends in Immunology 30: 513–521. 19699684 10.1016/j.it.2009.07.011

[16] Pilsczek FH, Salina D, Poon KKH, Fahey C, Yipp BG, et al (2010) A Novel Mechanism of Rapid Nuclear Neutrophil Extracellular Trap Formation in Response to *Staphylococcus aureus* . The Journal of Immunology 185: 7413–7425. 21098229 10.4049/jimmunol.1000675

[17] Urban CF, Reichard U, Brinkmann V, Zychlinsky A (2006) Neutrophil extracellular traps capture and kill *Candida albicans* yeast and hyphal forms. Cellular Microbiology 8: 668–676. 16548892 10.1111/j.1462-5822.2005.00659.x

[18] Bruns S, Kniemeyer O, Hasenberg M, Aimanianda V, Nietzsche S, et al (2010) Production of Extracellular Traps against *Aspergillus fumigatus In Vitro* and in Infected Lung Tissue Is Dependent on Invading Neutrophils and Influenced by Hydrophobin RodA. PLoS Pathog 6: e1000873. 20442864 10.1371/journal.ppat.1000873 PMC2861696

[19] Guimarães-Costa AB, Nascimento MTC, Froment GS, Soares RPP, Morgado FN, et al (2009) *Leishmania amazonensis* promastigotes induce and are killed by neutrophil extracellular traps. Proceedings of the National Academy of Sciences 106: 6748–6753. 10.1073/pnas.0900226106 PMC2672475 19346483

[20] Behrendt JH, Ruiz A, Zahner H, Taubert A, Hermosilla C (2010) Neutrophil extracellular trap formation as innate immune reactions against the apicomplexan parasite *Eimeria bovis* . Veterinary Immunology and Immunopathology 133: 1–8. 19625090 10.1016/j.vetimm.2009.06.012

[21] Fuchs TA, Abed U, Goosmann C, Hurwitz R, Schulze I, et al (2007) Novel cell death program leads to neutrophil extracellular traps. The Journal of Cell Biology 176: 231–241. 17210947 10.1083/jcb.200606027 PMC2063942

[22] Yousefi S, Mihalache C, Kozlowski E, Schmid I, Simon HU (2009) Viable neutrophils release mitochondrial DNA to form neutrophil extracellular traps. Cell Death Differ 16: 1438–1444. 19609275 10.1038/cdd.2009.96

[23] Remijsen Q, Berghe TV, Wirawan E, Asselbergh B, Parthoens E, et al (2010) Neutrophil extracellular trap cell death requires both autophagy and superoxide generation. Cell Res 21: 290–304. 21060338 10.1038/cr.2010.150 PMC3193439

[24] Bianchi M, Hakkim A, Brinkmann V, Siler U, Seger RA, et al (2009) Restoration of NET formation by gene therapy in CGD controls aspergillosis. Blood 114: 2619–2622. 19541821 10.1182/blood-2009-05-221606 PMC2756123

[25] Gabriel C, McMaster WR, Girard D, Descoteaux A (2010) *Leishmania donovani* Promastigotes Evade the Antimicrobial Activity of Neutrophil Extracellular Traps. The Journal of Immunology 185: 4319–4327. 20826753 10.4049/jimmunol.1000893

[26] Byrd AS, O’Brien XM, Johnson CM, Lavigne LM, Reichner JS (2013) An Extracellular Matrix–Based Mechanism of Rapid Neutrophil Extracellular Trap Formation in Response to *Candida albicans* . The Journal of Immunology 190: 4136–4148. 23509360 10.4049/jimmunol.1202671 PMC3622194

[27] Parker H, Dragunow M, Hampton MB, Kettle AJ, Winterbourn CC (2012) Requirements for NADPH oxidase and myeloperoxidase in neutrophil extracellular trap formation differ depending on the stimulus. Journal of Leukocyte Biology 92: 841–849. 22802447 10.1189/jlb.1211601

[28] Mohanan S, Horibata S, McElwee JL, Dannenberg AJ, Coonrod SA (2013) Identification of macrophage extracellular trap-like structures in mammary gland adipose tissue: a preliminary study. Frontiers in Immunology 4. 10.3389/fimmu.2013.00067 PMC3600535 23508122

[29] Aulik NA, Hellenbrand KM, Czuprynski CJ (2012) *Mannheimia haemolytica* and its leukotoxin causes macrophage extracellular trap (MET) formation by bovine macrophages. Infection and Immunity 80: 1923–1933. 22354029 10.1128/IAI.06120-11 PMC3347434

[30] Hellenbrand KM, Forsythe KM, Rivera-Rivas JJ, Czuprynski CJ, Aulik NA (2013) *Histophilus somni* causes extracellular trap formation by bovine neutrophils and macrophages. Microbial Pathogenesis 54: 67–75. 23022668 10.1016/j.micpath.2012.09.007 PMC7125803

[31] Wong K-W, Jacobs WR (2013) *Mycobacterium tuberculosis* Exploits Human Interferon γ to Stimulate Macrophage Extracellular Trap Formation and Necrosis. Journal of Infectious Diseases 208: 109–119. 23475311 10.1093/infdis/jit097 PMC3666134

[32] Menegazzi R, Decleva E, Dri P (2012) Killing by neutrophil extracellular traps: fact or folklore? Blood 119: 1214–1216. 22210873 10.1182/blood-2011-07-364604

[33] Banning N, Toze S, Mee BJ (2002) *Escherichia coli* survival in groundwater and effluent measured using a combination of propidium iodide and the green fluorescent protein. Journal of Applied Microbiology 93: 69–76. 12067376 10.1046/j.1365-2672.2002.01670.x

[34] Alby K, Schaefer D, Sherwood RK, Jones SK, Bennett RJ (2010) Identification of a Cell Death Pathway in *Candida albicans* during the Response to Pheromone. Eukaryotic Cell 9: 1690–1701. 20870881 10.1128/EC.00155-10 PMC2976293

[35] Young RL, Malcolm KC, Kret JE, Caceres SM, Poch KR, et al (2011) Neutrophil Extracellular Trap (NET)-Mediated Killing of *Pseudomonas aeruginosa*: Evidence of Acquired Resistance within the CF Airway, Independent of CFTR. PLoS ONE 6: e23637. 21909403 10.1371/journal.pone.0023637 PMC3164657

[36] Rose FRAJ, Bailey K, Keyte JW, Chan WC, Greenwood D, et al (1998) Potential Role of Epithelial Cell-Derived Histone H1 Proteins in Innate Antimicrobial Defense in the Human Gastrointestinal Tract. Infection and Immunity 66: 3255–3263. 9632593 10.1128/iai.66.7.3255-3263.1998 PMC108340

[37] Brinkmann V, Zychlinsky A (2007) Beneficial suicide: why neutrophils die to make NETs. Nat Rev Micro 5: 577–582. 10.1038/nrmicro1710 17632569

[38] McCormick A, Heesemann L, Wagener J, Marcos V, Hartl D, et al (2010) NETs formed by human neutrophils inhibit growth of the pathogenic mold *Aspergillus fumigatus* . Microbes and Infection 12: 928–936. 20603224 10.1016/j.micinf.2010.06.009

[39] Mège J-L, Mehraj V, Capo C (2011) Macrophage Polarization and Bacterial Infections. Current Opinion in Infectious Diseases 24: 230–234. 21311324 10.1097/QCO.0b013e328344b73e

[40] Charles A, Janeway Jr PT, Mark Walport, Shlomchik MJ, editors (2001) Immunobiology: The Immune System in Health and Disease. 5th edition. ed. New York: Garland Science.

[41] Gow NAR, van de Veerdonk FL, Brown AJP, Netea MG (2012) *Candida albicans* morphogenesis and host defence: discriminating invasion from colonization. Nat Rev Micro 10: 112–122. 10.1038/nrmicro2711 PMC3624162 22158429

[42] Akong-Moore K, Chow OA, von Köckritz-Blickwede M, Nizet V (2012) Influences of Chloride and Hypochlorite on Neutrophil Extracellular Trap Formation. PLoS ONE 7: e42984. 22912772 10.1371/journal.pone.0042984 PMC3418225

[43] Clark SR, Ma AC, Tavener SA, McDonald B, Goodarzi Z, et al (2007) Platelet TLR4 activates neutrophil extracellular traps to ensnare bacteria in septic blood. Nat Med 13: 463–469. 17384648 10.1038/nm1565

[44] Liu C, Tangsombatvisit S, Rosenberg J, Mandelbaum G, Gillespie E, et al (2012) Specific post-translational histone modifications of neutrophil extracellular traps as immunogens and potential targets of lupus autoantibodies. Arthritis Research & Therapy 14: R25. 22300536 10.1186/ar3707 PMC3392818

[45] Jann NJ, Schmaler M, Kristian SA, Radek KA, Gallo RL, et al (2009) Neutrophil antimicrobial defense against *Staphylococcus aureus* is mediated by phagolysosomal but not extracellular trap-associated cathelicidin. Journal of Leukocyte Biology 86: 1159–1169. 19638500 10.1189/jlb.0209053 PMC3192022

[46] Remijsen Q, Kuijpers TW, Wirawan E, Lippens S, Vandenabeele P, et al (2011) Dying for a cause: NETosis, mechanisms behind an antimicrobial cell death modality. Cell Death Differ 18: 581–588. 21293492 10.1038/cdd.2011.1 PMC3131909

[47] Tazzeo T, Worek F, Janssen LJ (2009) The NADPH oxidase inhibitor diphenyleneiodonium is also a potent inhibitor of cholinesterases and the internal Ca^2+^ pump. British Journal of Pharmacology 158: 790–796. 19788497 10.1111/j.1476-5381.2009.00394.x PMC2765598

[48] Dodd-o JM, Zheng G, Silverman HS, Lakatta EG, Ziegelstein RC (1997) Endothelium-independent relaxation of aortic rings by the nitric oxide synthase inhibitor diphenyleneiodonium. British Journal of Pharmacology 120: 857–864. 9138692 10.1038/sj.bjp.0701014 PMC1564554

[49] Hosseinzadeh A, Messer PK, Urban CF (2012) Stable redox-cycling nitroxide Tempol inhibits NET formation. Frontiers in Immunology 3. 10.3389/fimmu.2012.00391 PMC3529397 23269921

[50] Aulik NA, Hellenbrand KM, Klos H, Czuprynski CJ (2010) *Mannheimia haemolytica* and Its Leukotoxin Cause Neutrophil Extracellular Trap Formation by Bovine Neutrophils. Infection and Immunity 78: 4454–4466. 20823211 10.1128/IAI.00840-10 PMC2976348

[51] Hellenbrand KM, Forsythe KM, Rivera-Rivas JJ, Czuprynski CJ, Aulik NA (2013) *Histophilus somni* causes extracellular trap formation by bovine neutrophils and macrophages. Microbial Pathogenesis 54: 67–75. 23022668 10.1016/j.micpath.2012.09.007 PMC7125803

[52] Lin AM, Rubin CJ, Khandpur R, Wang JY, Riblett M, et al (2011) Mast Cells and Neutrophils Release IL-17 through Extracellular Trap Formation in Psoriasis. The Journal of Immunology 187: 490–500. 21606249 10.4049/jimmunol.1100123 PMC3119764

[53] Radic M, Marion T (2013) Neutrophil extracellular chromatin traps connect innate immune response to autoimmunity. Seminars in Immunopathology 35: 465–480. 23595413 10.1007/s00281-013-0376-6

[54] Kaplan MJ, Radic M (2012) Neutrophil Extracellular Traps: Double-Edged Swords of Innate Immunity. The Journal of Immunology 189: 2689–2695. 22956760 10.4049/jimmunol.1201719 PMC3439169

