# Supplementary figures and images for: Escherichia coli and Candida albicans Induced Macrophage Extracellular Trap-Like Structures with Limited Microbicidal Activity
Source: PLoS One. 2014 Feb 25;9(2):e90042. doi: 10.1371/journal.pone.0090042 (PMC3934966; doi:10.1371/journal.pone.0090042)

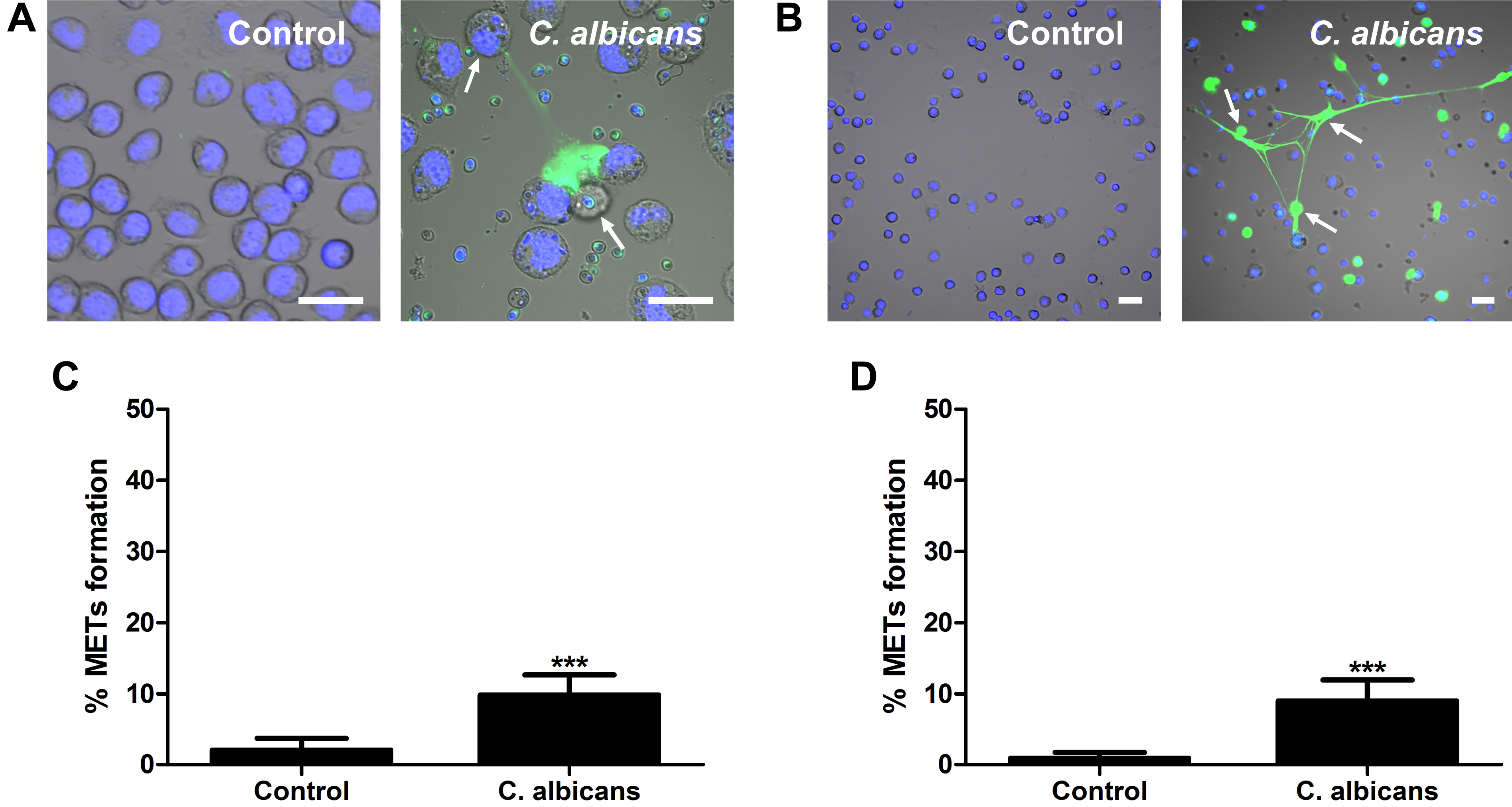

Supplement: Figure S1 — C. albicans yeast-form induces murine METs-LS formation. A-B: Murine J774A.1 macrophages (A) or peritoneal macrophages (B) were infected with C. albicans (MOI 1∶1) or a vehicle control and cultured at 30°C for 180 min. Hoechst 33342 (Blue) and SYTOX Green (Green) were added to label the nucleus and extracellular DNA. The arrows indicate macrophages releasing METs-LS in response to the C. albicans yeast-form. Scale Bars: 20 µm. C–D: The quantification of METs-LS-positive cells in murine J774A.1 macrophages (C) or peritoneal macrophages (D) stimulated with the C. albicans yeast-form, the result is shown as the means ± SD (n = 5). ***P<0.001 compared with the control group by two tailed Student’s t-test, respectively. The experiments were repeated independently 3 times with similar results. (TIF) [file pone.0090042.s001.tif]

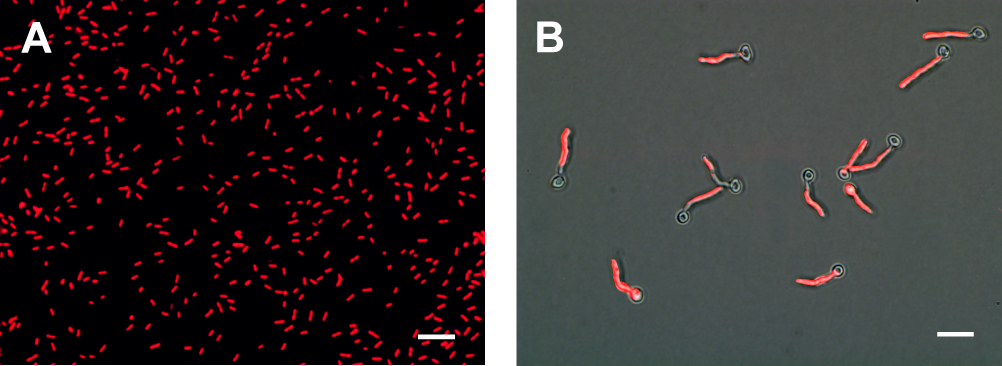

Supplement: Figure S2 — Dead E. coli and C. albicans are stained red by PI. E. coli treated with 200 µg/ml streptomycin sulfate for 2 h (A) and C. albicans treated at 100°C for 15 min (B) were used as positive control for PI staining. Scale Bars: 10 µm. (TIF) [file pone.0090042.s002.tif]

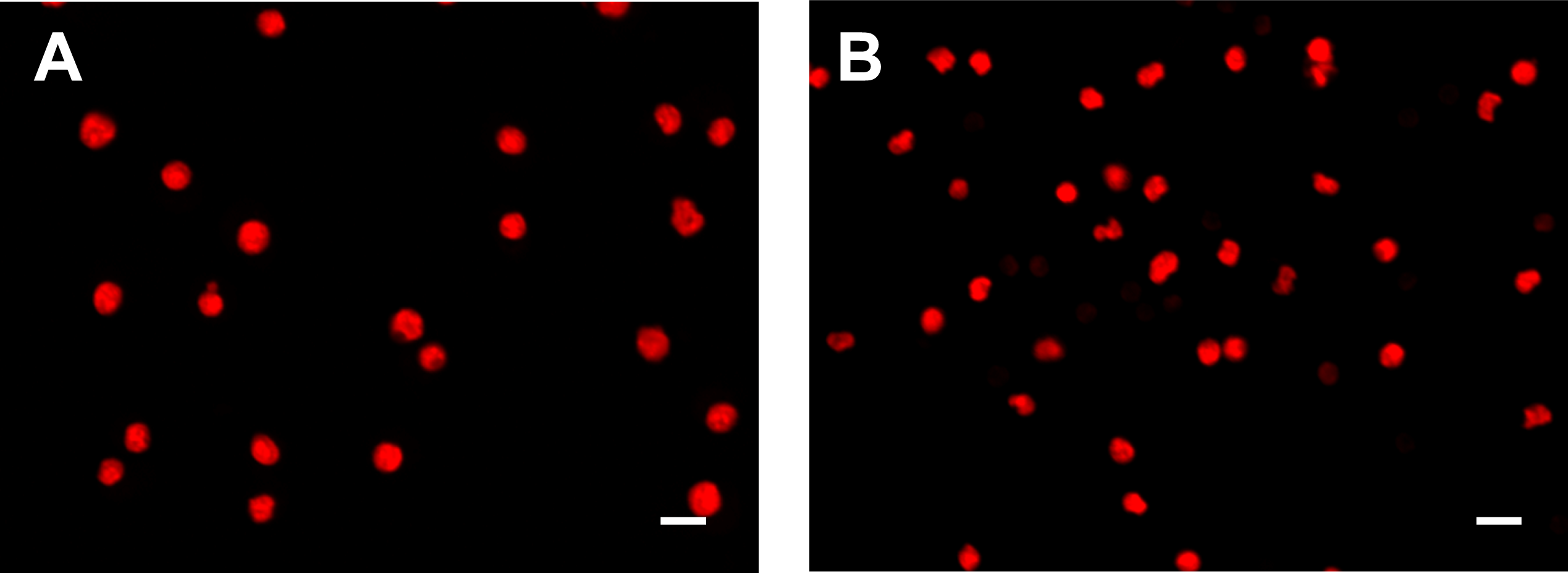

Supplement: Figure S3 — Necrotic macrophages do not form METs-LS. PI staining of necrotic J774A.1 macrophages (A) and necrotic peritoneal macrophages (B) induced by hydrogen peroxide (100 µM, 3 h). Scale Bars: 20 µm. (TIF) [file pone.0090042.s003.tif]
